# 3D human pose point cloud data of light detection and ranging (LiDAR)

**DOI:** 10.1016/j.dib.2025.112043

**Published:** 2025-09-10

**Authors:** Farah Zakiyah Rahmanti, Moch. Iskandar Riansyah, Oddy Virgantara Putra, Eko Mulyanto Yuniarno, Mauridhi Hery Purnomo

**Affiliations:** a Department of Electrical Engineering, Institut Teknologi Sepuluh Nopember, Surabaya, 60111, Indonesia; b Department of Information Technology, Telkom University, Surabaya Campus, Surabaya, 60231, Indonesia; c Department of Electrical Engineering, Telkom University, Surabaya Campus, Surabaya, 60231, Indonesia; d Department of Informatics, Universitas Darussalam Gontor, Ponorogo, 63472, Indonesia; e Department of Computer Engineering, Institut Teknologi Sepuluh Nopember, Surabaya, 60111, Indonesia

**Keywords:** 3D LiDAR, Human pose prediction, Spatial, Geometric, Temporal, 3D point cloud dataset

## Abstract

3D Light Detection and Ranging (LiDAR) sensors are closely related to computer vision and deep learning. 3D LiDAR sensors are commonly embedded in smart vehicles to segment humans, cars, trucks, motors, and other objects. However, 3D LiDAR can also be used indoors to predict human poses that are more friendly to a person's privacy because 3D LiDAR does not capture facial images, but it produces data in the form of point clouds. The point cloud produces spatial, geometric, and temporal information which can be used to predict, detect, and classify human poses and activities. The data output from 3D LiDAR, which includes spatial and temporal data, is in PCAP (.pcap) and JSON (.json) formats. The PCAP file contains the sequence frame of the 3D human pose point cloud, and the JSON file contains the metadata. Each human pose class label has one PCAP file and one JSON file. The raw spatio-temporal data must be processed into PCD format as a 3D human pose point cloud dataset for each human pose.

The total human pose dataset is 1400 3D point cloud data with PCD format (.pcd) used for the training and testing process in deep learning, consisting of four human pose labels. The label classes are hands-to-the-side, sit-down, squat-down, and stand-up human poses, with each class having 280 3D point cloud data used as training data. While the test data amounted to 280 3D point cloud data. The data collection process uses 3D LiDAR, a tripod, a personal computer/laptop, and a talent, demonstrating basic human poses. The 3D LiDAR used is OS1, a product of Ouster, which has a range of 90–200 m, 128 channels of resolution, and a temperature of -40 – 60° C. For talent, there is one person and male gender in this current shooting. However, in its development, it can also take female or children or elderly talent to enrich the human pose dataset. The talent is between 30 and 40 years old. The distance between the 3D LiDAR and the talent position is 120 cm. Data collection took place from 10:00 a.m. to 1:00 pm. indoors.

This dataset is used for human pose prediction using one of the deep learning algorithms, Convolutional Neural Network (CNN). However, the developers can also use other deep learning algorithms such as transformers, Graph Neural Network (GNN), etc.

Specifications Table

**Table:** 

Subject	Computer Sciences
Specific subject area	3D human pose point cloud dataset of Light Detection and Ranging (LiDAR).
Type of data	Type of data: Raw and has been processed Data formats: pcap, json, pcd
Data collection	Our dataset was compiled utilizing a single person as a talent demonstrating several basic human poses using a 3D LiDAR sensor. These basic human poses include hands-to-the-side, sit-down, squat-down, and stand-up, which are also class labels. The data collection process uses a 3D LiDAR, a tripod, a personal computer/laptop, and a talent. The minimum specification of a personal computer is a CPU Intel(R) Core (TM) i5–8350 U CPU @1.70 GHz and memory 16GB speed 2400 MHz. The distance between the 3D LiDAR and the talent position is about 120 cm. The data output of 3D LiDAR, which includes spatial and temporal data, is in PCAP (.pcap) and JSON (.json) formats. This raw data needs to be human segmented using 3D slicing to obtain human Regions of Interest (ROI) and manual labelling for each segment and ensure accuracy and ease of use, so that it can be applied to decision-making. The minimum software required for this process is PyCharm Community Edition 2022.2.1, but it can be replaced with other software, such as Visual Studio Code, etc. The programming language used is Python and the library used to visualize point cloud data is open3d. The python code for performing the conversion PCAP to PCD (pcap2pcd) can be seen in the GitHub link provided with the file name pcap2pcd.py. The Python conversion pcap2pcd is also in our Mendeley Data page parts step to reproduce. The developer can do normalization of 3D points using the min-max scaler method before starting to the learning process with deep learning algorithms.
Data source location	Geographical Coordinates: −7.282662,112.7951935 Location: Laboratorium of Multimedia Internet of Things, Department of Electrical Engineering Institution: Institut Teknologi Sepuluh Nopember City/Town/Region: Surabaya, East Java, 60,115 Country: Indonesia
Data accessibility	Repository name: Mendeley Data Data identification number: doi:10.17632/gpvrnphw66.3 Direct URL to data: https://data.mendeley.com/datasets/gpvrnphw66/3
Related research article	[1]

## 1 Value of the Data

• Spatial Information: This dataset was obtained from a 3D LiDAR sensor, which generates 3D point cloud data, which has information in 3D space. Each point of the human pose is represented in 3D coordinates (x, y, z). This helpful information can be the first step in a feature extraction approach based on points, voxels, graphs, etc. Furthermore, the spatial information includes the relative positioning and the distance. This spatial information is also used in the denoising and the normalization steps before starting the learning process using machine learning or deep learning algorithms.
• Geometric Information: a 3D point cloud shows the geometric structure of the human body, such as the body joints, poses, postures, size, orientation, topology, and position in 3D space. The dataset provides geometric information in the form of 3D points that show the position of the head, hands, or feet in 3D space, body size, body orientation facing forward or vice versa, and the distance between the points so that it can distinguish several different human poses. The computer vision application which utilizes geometric information, such as obtaining the skeleton of the human body, human pose prediction, and human pose estimation.
• Temporal Information: 3D LiDAR scans objects and captures changes in object movement in a few moments, depending on the scanning time. The dataset provides temporal information in the form of time sequences, durations, and transitions of human poses in 3D space. This potential information can help recognize human pose estimation, human pose tracking, and human activities such as basic activities (stand-up, sit-down, squat-down) and exercising (hands to the side). The human pose was chosen because it is basic for many human activities.
• Health Field: Track movement by analyzing human pose for physiotherapy medic which focuses on restoring and improving the body's function. Human pose analysis by professional medical personnel can help provide appropriate recommendations for recovery patients, especially in physical and movement. This dataset can be used as a benchmark for healthy adult human poses, as there are fundamental differences between healthy and unhealthy human poses. However, this dataset only provides human poses for healthy adults.
• Monitoring application and safety analytics for anomaly detection: There are several human tracking applications that can be used for various topics, such as pose estimation and gesture recognition, human activity recognition, elderly monitoring from early fall detection, near-fall detection, exercising, and occupational safety by detecting abnormal human poses. However, dataset collection will continue to be improved by the development of research conducted and will not only focus on basic human poses. Potential deployments in the bathroom or bedroom, which prioritize privacy.
• The dataset supports anomaly detection under challenging conditions, such as low illumination or backlighting, whereas the reliability of 3D LiDAR is.
• Human Pose Prediction, Computer Vision, and Deep Learning: This dataset is invaluable for researchers and developers in human pose prediction, computer vision, and deep learning. It provides an opportunity to develop deep learning models that aim to understand human poses and movements. Computer vision and deep learning development can be combined with other sensors like mmWave radar, camera, etc. Multimodal can be implemented in several computer vision and deep learning case studies.

## 2 Background

The development of computer vision-based technology utilizing 3D LiDAR sensors offers spatial, geometric, and temporal information that has the potential to predict and analyze human poses, postures, movements, orientations, and activities with greater accuracy. The availability of public 3D point cloud datasets of humans indoors is still minimal. This dataset contributes to the development of innovations in computer vision and deep learning related to human pose prediction [1], human orientation [2], denoising human pose [3], object classification [3,4], human pose estimation, and human activities, among others. For example, innovations in the health sector, such as physiotherapy and medical rehabilitation, sports, human-computer interaction, and security, have been developed. This 3D human pose point cloud dataset aims to engage a wider audience, including developers and the public. This dataset serves as a means of interdisciplinary research and community service, bridging medical staff with the latest technology and connecting sports coaches with the latest innovations.

Table 1 shows a comparative overview of several previous datasets. The differences are visible based on the dataset's value, the device used in data collection, limitations, and the dataset gap. Our valuable datasets are privacy-preserving human sensing and spatio-temporal 3D point cloud data.

**Table 1:** Comparative Overview of Different Dataset.

Dataset	Value	Device used	Limitation	Gap
[5]	Dataset for Emergency Medical Services (EMS) vehicles	LiDAR and RGB camera	EMS vehicles, Created using the CARLA simulator	Outdoor area and focused on enriching specialized objects such as EMS vehicles
[6]	Privacy-Preserving Human Sensing	Radar and RGB-D camera	Supports 3D pose estimation and gesture recognition	Radar produces sparser data, pose details may be lost
[7]	Dataset with high-density point cloud	MOVE4D System	Does not contain temporal data	Focused on a snapshot per pose
[8]	Sequence frame of Video	Digital camera 12 MP	Does not privacy-preserving human sensing	A different way of data collection and focused on 2D data per frame
Current	Sequence frame of 3D human pose point cloud, Privacy-preserving human sensing, spatial and temporal 3D point cloud data	3D LiDAR	One male as a talent, Indoor area, specific human pose	Focused on 3D point cloud data and human pose

## 3 Data Description

**Fig. 1:**
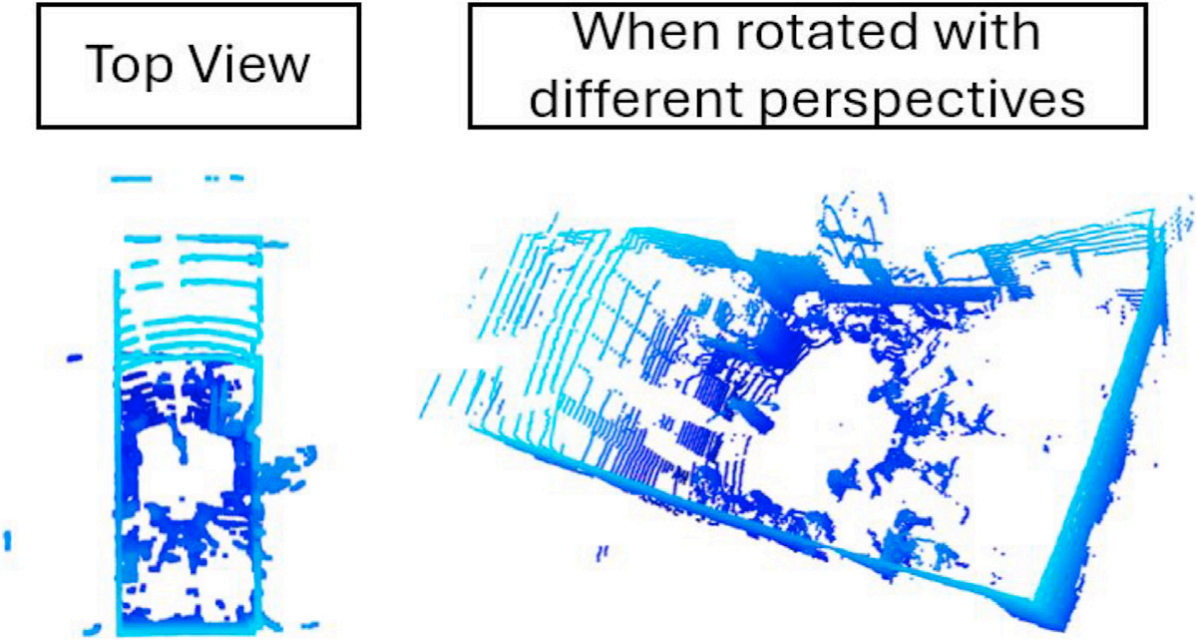
Raw Data 3D Point Cloud of LiDAR.

**Fig. 2:**
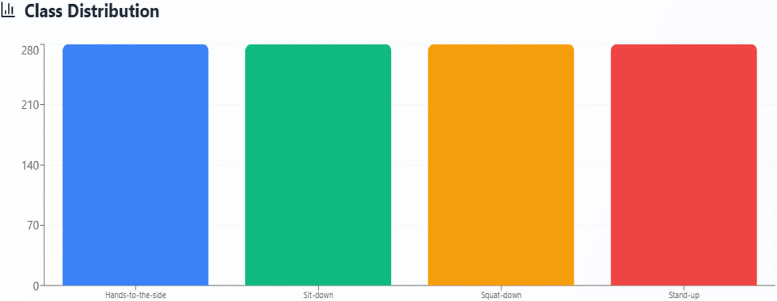
Balance Data based on Number of Datasets.

The data output from the 3D LiDAR sensor is in PCAP (.pcap) and JSON (.json) formats as raw data. The form of data is spatio-temporal point cloud data, one frame 3D point cloud generated of 3D LiDAR as in Fig 1. The raw data needs to be segmented to obtain a 3D human pose point cloud that only focuses on humans. The human segmentation process produces a total of 1400 3D point cloud data according to Table 2 with the class distribution according to Fig 2. Each human pose class has 280 data, the total data contained in the four classes is 1120 data points. The proportion between training and testing data is 80 % and 20 %. The amount of test data used is 280 data.

**Table 2:** Dataset Allocation.

Num	Data Class	Number of Data (3D point cloud)	Allocation
1	Hands-to-the-side	280	Training Data
2	Sit-down	280	Training Data
3	Squat-down	280	Training Data
4	Stand-up	280	Training Data
5	(Mix Human Poses: hands-to-the-side, sit-down, squat-down, and stand-up)	280	Testing Data
	**Total**	**1400**	-

The dataset owned includes balanced data with the same amount in each class. The advantage of the balance dataset is that the deep learning model will learn fairly for all human pose classes, thereby reducing the risk of bias towards certain classes and minimizing special handling of datasets such as resampling.

The dimensions of length, width, and height of 3D human poses vary. This dimension depends on the poses; for example, a standing pose will have different dimensions than a pose with hands to the side. Despite the differences in size in 3D space, this proves that the existing dataset has challenges in processing the data. This dataset will be particularly useful for developers and researchers in computer vision, deep learning, health, and sports who are interested in pose, posture, and body orientation. The dataset offers opportunities for anyone seeking to understand, explore, and process data using point cloud approaches and deep learning methods.

## 4 Experimental Design, Materials and Methods

The minimum specification of a personal computer is a CPU Intel(R) Core (TM) i5–8350 U CPU @1.70 GHz and memory 16GB speed 2400 MHz. The specification device used is the 3D LiDAR sensor used is OS1 is shown on Fig 5, a product of Ouster which has a range of 90–200 m, 128 channels of resolution, and a temperature of −40 – 60° C. The minimum software required for this process is PyCharm Community Edition 2022.2.1, but it can be replaced with other software such as Visual Studio Code, etc.

The data output from 3D LiDAR, which includes spatial and temporal data, is in PCAP (.pcap) and JSON (.json) formats. Each human pose class label has one PCAP file and one JSON file. The raw spatio-temporal data must be processed into PCD format as a 3D human pose point cloud dataset for each human pose.

Some examples of human pose visualization on 3D point cloud data using the Open3D library are shown in Table 3. The visualization of each pose is based on three representative methods: scanning with the body facing forward, backward, and sideways using 3D LiDAR. The visualization of each scanning is based on the front view, side view, back view, and top view.

**Table 3:** Example of 3D Human Pose Point Cloud Dataset.

**Fig. 3:**
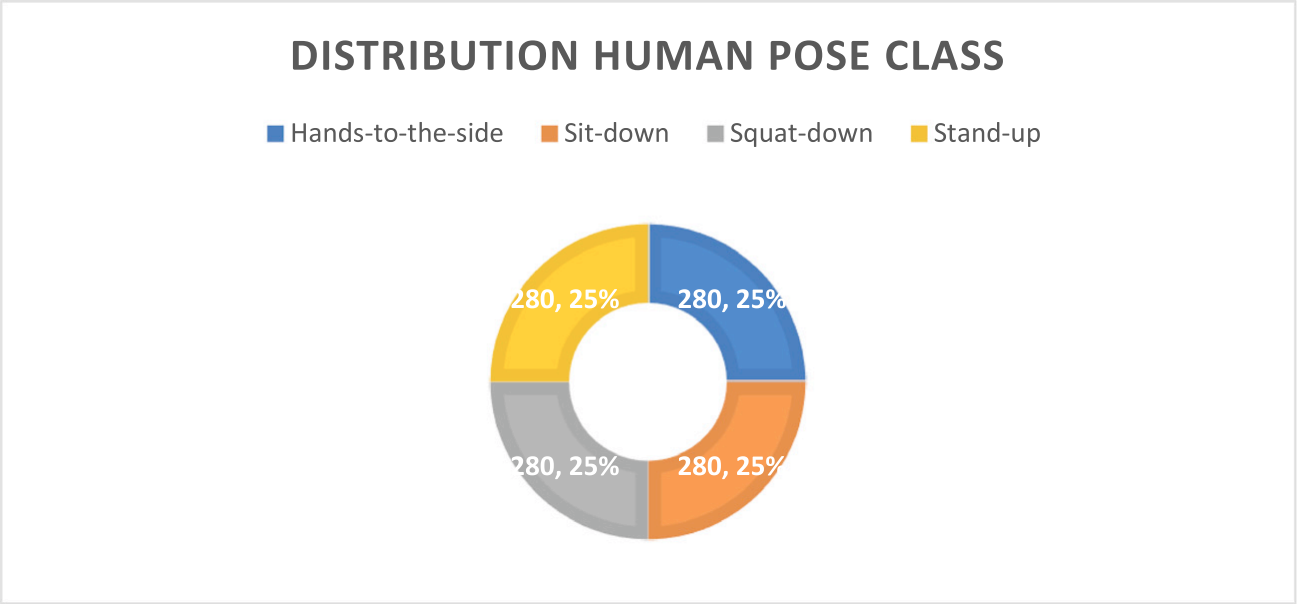
Distribution of Human Pose Class Label.

**Fig. 4:**
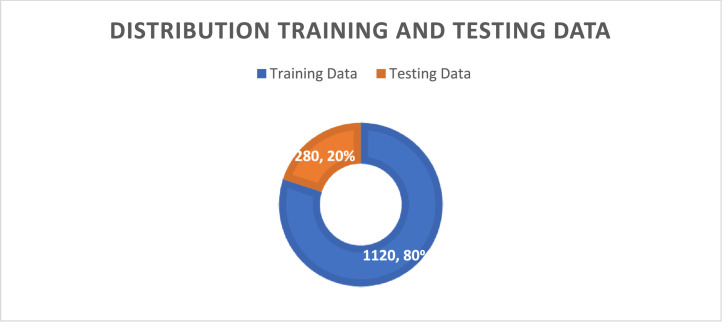
Distribution of Training and Testing Data.

The total dataset consists of 1400 3D human point cloud data, where 1120 are training data and 280 are testing data. Each class consists of 280 data, according to Table 2. The distribution of the dataset for each class is shown on Fig 3. Each class comprises 25 % of the training data. According to Fig 4, the distribution of the training data and testing data is as follows: the training data has a distribution of 80 %, and the testing data has a distribution of 20 %.

**Fig. 5:**
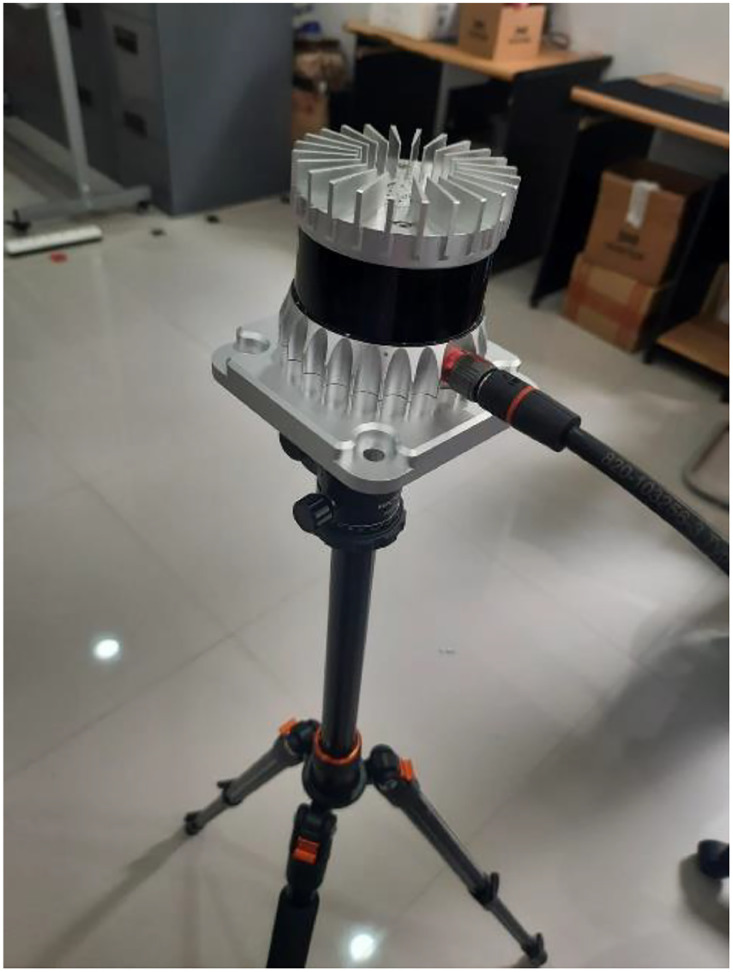
OS1 3D LiDAR Sensor.

**Fig. 6:**
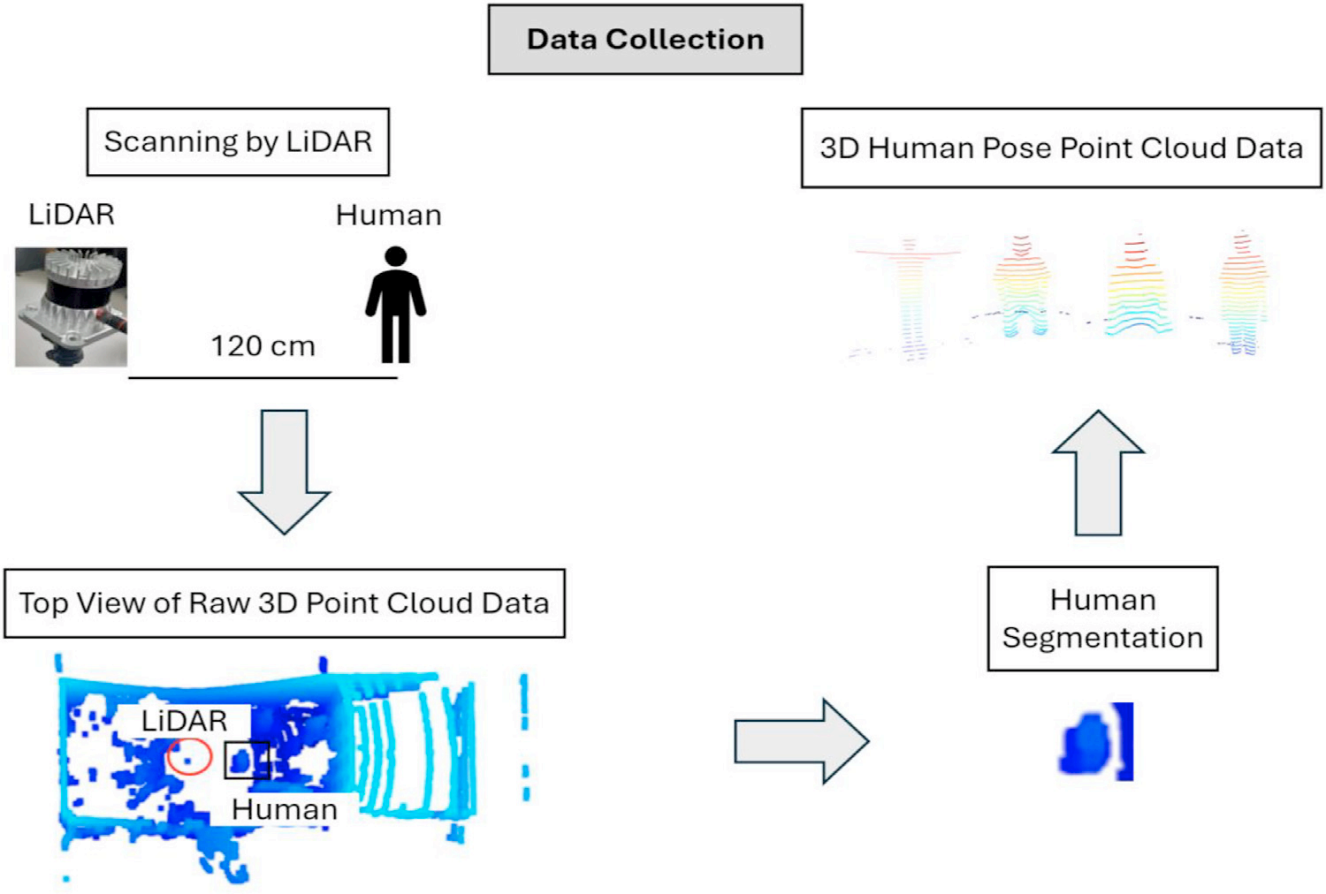
Data Collection.

The data collection process is shown in Fig 6 3D LiDAR scans at a distance of 120 cm from humans. The three important things here are getting the file source PCAP with pcap.Pcap(pcap_path, info) and also this meta data source.metadata, the last is scans to convert PCAP and get PCD per frame with client.Scans(source). Import pcap and client from library ouster. The python code for performing the conversion pcap2pcd can be seen in the GitHub link provided with the file name pcap2pcd.py. The Python conversion pcap2pcd is also in our Mendeley Data page parts step to reproduce.

Raw data from 3D LiDAR is visualized using Python and open3D. The visualization results can be viewed from various directions, for example, top view, side view, or by rotating; it will get a view with a different perspective. The following process is human segmentation, which is done by cropping 3D to identify the region of interest. The final process is labeling and adjusting each 3D human point cloud data to its human pose label class.

## Limitations

The dataset has significant limitations, including only representing four basic human poses, the talent involved is one person, and data collection is only indoors. The talent is between 30 and 40 years old. The distance between the 3D LiDAR position and the talent position is 120 cm. Data collection took place from 10:00 a.m. to 1:00 pm. indoors. Our work also considers the early study of human pose prediction. Some improvements in data collection are needed, such as taking other human poses, diverse talent, and multi-person, and data collection is also carried out outdoors. More varied human poses and diverse talent from various sexes (male or female) and ages (children, adults, or the elderly) will add value to the data. Our limitations will impact the diversity of human body shapes and movements later. These highlight limitations for more diverse data collection in the real world, considering the correct pre-processing method based on noise factor will affect the results of the human pose prediction.

## Ethics Statement

This research complies with ethical standards because the data is in the form of a point cloud that does not display an image of a person and does not display personal information.

## Credit Author Statement

**Farah Zakiyah Rahmanti:** Software, Methodology, Formal analysis, Resources, Data curation, Writing –original draft, Writing –review & editing, Visualization; **Moch. Iskandar Riansyah:** Software, Data curation, Investigation, Writing –review & editing; **Oddy Virgantara Putra:** Software, Data curation, Investigation, Writing –review & editing; **Eko Mulyanto Yuniarno:** Conceptualization, Methodology, Validation, Resources, Supervision, Project administration; **Mauridhi Hery Purnomo:** Conceptualization, Validation, Resources, Supervision, Funding acquisition.

## Data Availability

Mendeley Data3D Human Pose Point Cloud Data of Light Detection and Ranging (LiDAR) (Original data)
Mendeley Data3D Human Pose Point Cloud Dataset of Light Detection and Ranging (LiDAR) (Original data)
